# An AML1-ETO/miR-29b-1 regulatory circuit modulates phenotypic properties of acute myeloid leukemia cells

**DOI:** 10.18632/oncotarget.18127

**Published:** 2017-05-24

**Authors:** Sayyed K. Zaidi, Andrew W. Perez, Elizabeth S. White, Jane B. Lian, Janet L. Stein, Gary S. Stein

**Affiliations:** ^1^ Department of Biochemistry and University of Vermont Cancer Center, The University of Vermont Larner College of Medicine, Burlington, VT, USA

**Keywords:** RUNX, leukemia, miR-29, AML1-ETO, t(8;21)

## Abstract

Acute myeloid leukemia (AML) is characterized by an aggressive clinical course and frequent cytogenetic abnormalities that include specific chromosomal translocations. The 8;21 chromosomal rearrangement disrupts the key hematopoietic RUNX1 transcription factor, and contributes to leukemia through recruitment of co-repressor complexes to RUNX1 target genes, altered subnuclear localization, and deregulation of the myeloid gene regulatory program. However, a role of non-coding microRNAs (miRs) in t(8;21)-mediated leukemogenesis is minimally understood. We present evidence of an interplay between the tumor suppressor miR-29b-1 and the AML1-ETO (also designated RUNX1-RUNX1T1) oncogene that is encoded by the t(8;21). We find that AML1-ETO and corepressor NCoR co-occupy the miR-29a/b-1 locus and downregulate its expression in leukemia cells. Conversely, re-introduction of miR-29b-1 in leukemia cells expressing AML1-ETO causes significant downregulation at the protein level through direct targeting of the 3’ untranslated region of the chimeric transcript. Restoration of miR-29b-1 expression in leukemia cells results in decreased cell growth and increased apoptosis. The AML1-ETO-dependent differentiation block and transcriptional program are partially reversed by miR-29b-1. Our findings establish a novel regulatory circuit between the tumor-suppressive miR-29b-1 and the oncogenic AML1-ETO that controls the leukemic phenotype in t(8;21)-carrying acute myeloid leukemia.

## INTRODUCTION

Acute Myeloid Leukemia (AML) is an aggressive neoplasm with frequent cytogenetic abnormalities, a recurrent clinical course and a limited number of drug treatment options [1]. More than 25% of AML patients carry the 8;21 chromosomal rearrangement, which disrupts the gene encoding the essential hematopoietic RUNX1 transcription factor [2]-[4]. The *RUNX1* gene is required for definitive hematopoiesis, and is a frequent target of mutations and translocations in various leukemia types [3]. In normal myeloid cells, RUNX1 protein transcriptionally regulates genes essential for myeloid differentiation by interacting with promoter regulatory regions in a sequence specific manner via the amino-terminal DNA binding domain and recruiting coregulatory proteins for transcriptional activation or suppression via carboxy terminus [2], [5]. Importantly, RUNX1 is localized in punctate nuclear domains through a subnuclear targeting signal located in the carboxy terminus, and the intranuclear localization of RUNX1 is required for biological activity [6]-[8]. The 8;21 chromosomal translocation, which is prevalent in acute myeloid leukemia, combines the first 5 exons of the *RUNX1* gene, located on chromosome 21, with nearly all of the *ETO* gene, located on chromosome 8, and generates a chimeric transcript encoding the oncogenic AML1-ETO (also called RUNX1-RUNX1T1) protein [9], [10]. AML1-ETO protein retains the DNA binding domain of RUNX1, but the ETO moiety replaces the carboxy terminus that contains protein interaction domains necessary for normal functional activity, as well as the subnuclear targeting signal responsible for the punctate nuclear localization of RUNX1 regulatory complexes [10]-[12]. Consequently, AML1-ETO occupies and deregulates RUNX1 target genes, as well as localizes to subnuclear sites that are distinct from those where RUNX1 resides, thus resulting in leukemia phenotype [3], [11], [13]. Importantly, the chimeric transcript encoding the AML1-ETO oncogene carries the 3’UTR of the *ETO* gene that is distinct from that of the wild type *Runx1* RNA [14]. Because the ETO gene is not normally expressed in hematopoietic cells, specific targeting of its 3’UTR has potential therapeutic value in AML.

MicroRNA (miRs) regulate nearly all essential biological pathways by interacting with 3’ untranslated regions of transcripts and inhibiting their translation into corresponding proteins. MicroRNAs have the potential for both diagnosis and therapeutic intervention in cancer progression of solid tumors and leukemias and are a recent focus of intense investigation [15]-[19]. For example, several miRs that include miR-24, miR-125, miR-181, and miR-193 mechanistically regulate various steps of hematopoiesis and leukemogenesis [20]-[23]. Similarly, members of the miR-29 family are emerging as tumor suppressors in solid tumors and hematological malignancies [24], [25]. Of particular interest, expression of miR-29 family members, encoded by chromosomes 1 (miR-29b-2/c) and 7 (miR-29a/b-1), is downregulated in various leukemia subtypes, including AML [24], [26]. Some key transcriptional upregulators of miR-29 family members include SP1, RUNX3, and C/EBPa [27]-[29]. Mature miR-29 family members target proteins that are involved in key cellular processes in hematopoietic and leukemic cells including AKT2 [30], CDK6 [31], DNMT3A & B [32], ABL1 & BCR-ABL1 [33] and SP1 [34]. However, a role of miR-29 family members in t(8;21)-carrying AML has not been explored.

We demonstrate that miR-29b-1 targets the 3’UTR of the AML1-ETO oncogene. We present evidence that AML1-ETO and its corepressor NCoR co-occupy the miR-29a/b-1 locus and down-regulate its expression. Re-introduction of miR-29b-1 in leukemic cells expressing AML1-ETO causes significant downregulation at the protein level. Concomitantly, cells exhibit decreased cell growth and increased apoptosis. Furthermore, miR-29b-1 partially reverses the AML1-ETO-induced differentiation block and modifies the AML1-ETO-mediated transcriptional program. Together, our findings establish a novel regulatory circuit between the tumor-suppressive miR-29b-1 and the oncogenic AML1-ETO that controls the leukemic phenotype in t(8;21)-carrying acute myeloid leukemia.

## RESULTS

### AML1-ETO transcriptionally downregulates miR-29b-1, a miR that directly targets AML1-ETO protein in leukemic cells

**Figure 1:**
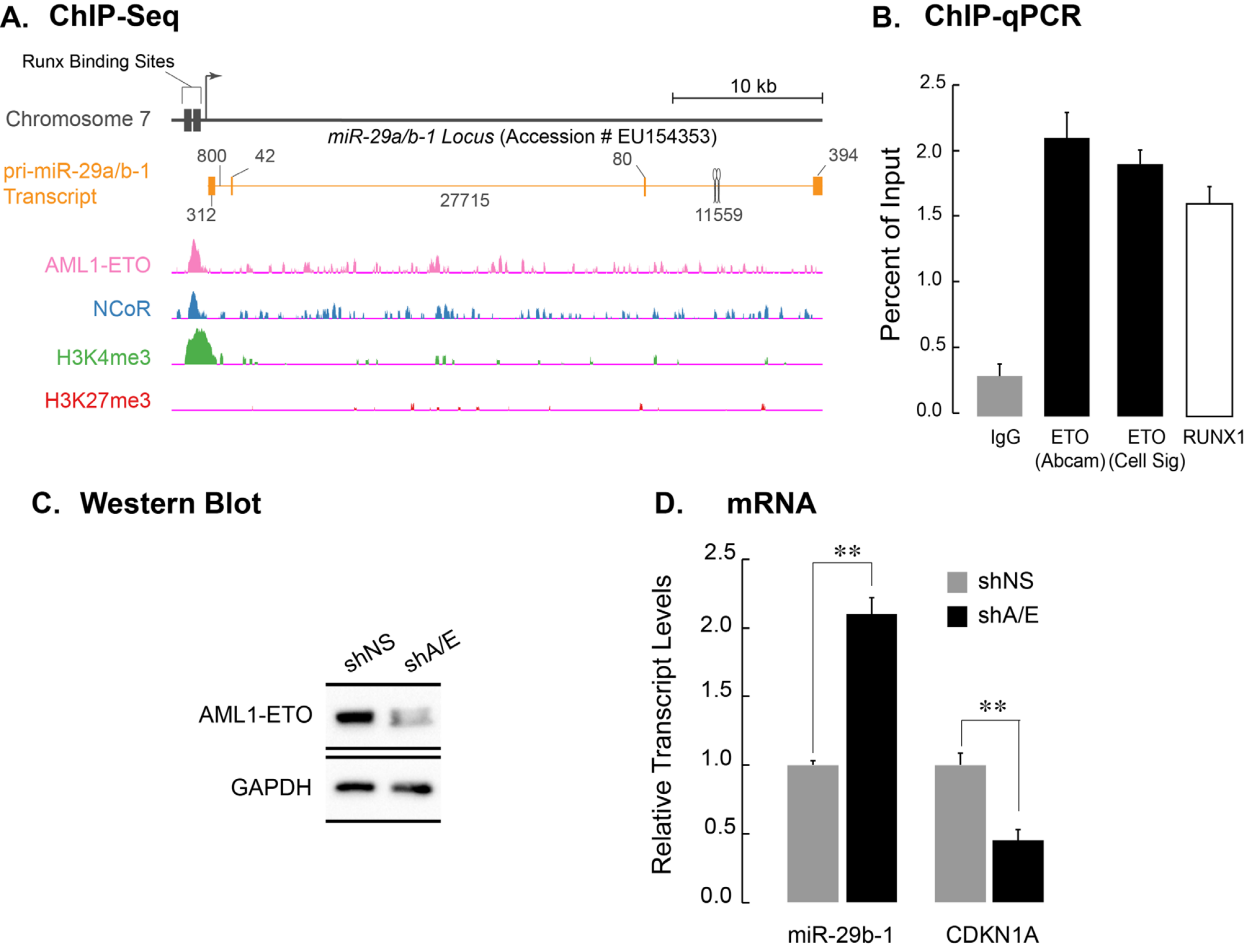
AML1-ETO occupies the miR-29a/b-1 locus and transcriptionally down regulates the miR-29a/b-1 primary transcript **A.** Top schematic shows the *miR-29a/b-1* locus on human chromosome 7 (Accession Number EU154353) [45]. Runx binding sites are indicated. RNAs originating from the locus are shown; orange blocks indicate exons, while the orange line shows intronic regions. Genomic tracks from ChIP-seq dataset from Trombly *et al* [35] were re-analyzed for occupancy of the miR-29a/b-1 locus by AML1-ETO, NCoR, or the activating H3K4me3 and suppressive H3K27me3 histone modifications. **B.** Actively proliferating Kasumi-1 cells were subjected to chromatin immunoprecipitation with two separate antibodies against ETO (black bars), a rabbit polyclonal antibody against carboxy-terminus of RUNX1, which is not present in AML1-ETO (white bar), and an IgG isotype control (gray bar). Immuno-enriched chromatin was analyzed by quantitative PCR using primers encompassing Runx binding sites in the regulatory region of the *miR-29a/b-1* locus. Bar graph shows immuno-enriched chromatin as a percentage of input. **C**. Western blot analysis of SKNO-1 cells infected with a non-silencing short hairpin RNA (shNS), or an shRNA selectively targeting AML1-ETO (shA/E). Significant knock down of the AML1-ETO protein was reproducibly obtained 5 days post-infection. GAPDH was used as protein loading control. **D.** Total cellular RNA from SKNO-1 cells infected with shNS and shA/E was analyzed for expression of the pri-miR-29a/b-1, as well as of *CDKN1A*, a known AML1-ETO target gene. Bar graph represents an average of three independent experiments, and error bars indicate standard deviation (SD). Student's t-test shows highly significant changes in the expression of pri-miR-29b-1 and *CDKN1A* (*p* > 0.01) upon AML1-ETO knockdown.

We have previously shown that genes co-occupied by AML1-ETO and its corepressor N-CoR are deregulated upon AML1-ETO depletion and contribute to the leukemia phenotype [35]. Furthermore, the *miR-29a/b-1* locus contains RUNX binding sites (Figure 1A) and is regulated by RUNX3, a RUNX family member that shares a highly conserved DNA binding domain with RUNX1 and AML1-ETO [27]. We therefore assessed whether the *miR-29a/b-1* locus is a part of AML1-ETO/N-CoR signature. Figure 1A shows genomic tracks of Kasumi-1 cells subjected to the chromatin immunoprecipitation-deep sequencing (ChIP-Seq) using antibodies against AML1-ETO, N-CoR, and the activating H3K4me3 or repressive H3K27me3 histone modifications. We find that the locus is co-occupied by both AML1-ETO and N-CoR, suggesting that miR-29b-1 is a key component of leukemia signature. We experimentally verified these findings using ChIP-qPCR in Kasumi-1 cells; antibodies specific for AML1-ETO (from two independent sources) and RUNX1 were used for immunoprecipitation and an isotype-matched normal IgG was used as control. The immuno-enriched chromatin was assayed for presence of the *miR-29b-1* locus by qPCR using a specific primer set surrounding the predicted RUNX binding sites. As shown in Figure 1B, the *miR-29a/b-1* locus was occupied by AML1-ETO (black bars) as well as by the wild type RUNX1 (white bars). As a positive control, we also used a primer set specific for the RUNX1 P1 promoter, which contains many RUNX binding sites and is occupied by AML1-ETO and RUNX1 [35]. As expected, RUNX1 P1 promoter was occupied by both AML1-ETO (black bars) and RUNX1 (white bars)(Supplementary Figure 1).

We addressed whether occupancy of the miR-29a/b-1 locus results in transcriptional regulation of the *miR-29a/b-1* gene by AML1-ETO. Kasumi-1 cells, which express AML1-ETO, were infected with lentivirus carrying short-hairpin RNA (shRNA) designed to specifically target AML1-ETO (shA/E); a non-silencing shRNA (shNS) was included as a control for specificity. Total protein and RNA were isolated from cells that were harvested 72 hours after infection. The shRNA-mediated down-regulation of AML1-ETO transcript and protein was assessed by qPCR and western blot analysis, respectively. As shown in Figure 1C, AML1-ETO protein was significantly downregulated when compared to a non-silencing control, although the effect of shRNA on transcript levels was less pronounced (data not shown). Conversely, expression of the primary transcript for miR-29a/b-1 was significantly up-regulated (more than 2 fold; *p* < 0.01). This result indicates that AML1-ETO down-regulates the *pri-miR-29b-1* transcript (Figure 1D). As a control, we also determined the expression of the *CDKN1A* gene, a known and direct target gene that is transcriptionally upregulated by AML1-ETO. As expected, the *CDKN1A* gene was down-regulated upon depletion of AML1-ETO (*p* > 0.01) (Figure 1D).

**Figure 2:**
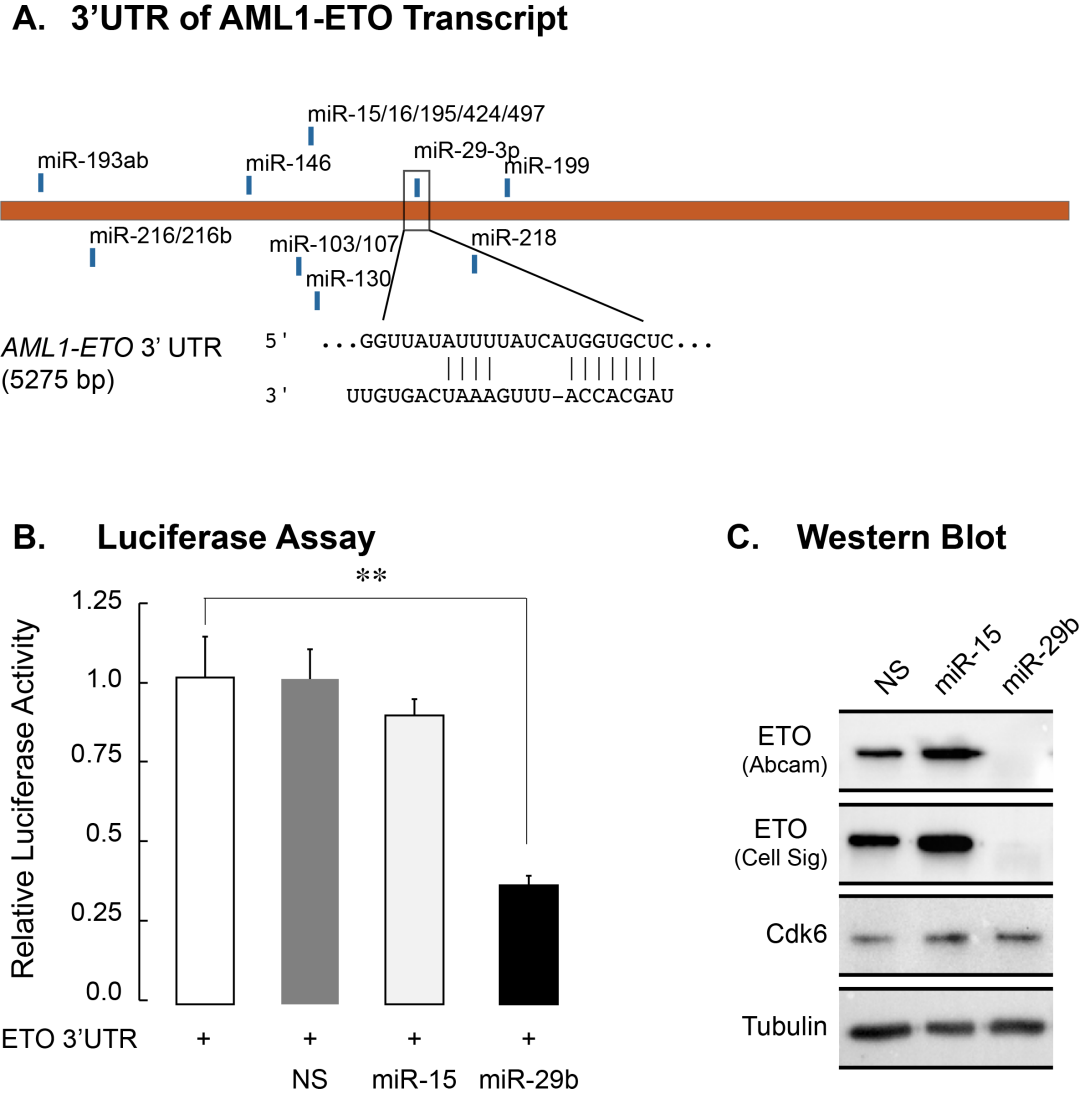
miR-29b-1 targets the 3’UTR of the AML1-ETO transcript and down-regulates AML1-ETO protein in leukemia cells **A.** The *ETO/RUNX1T1* transcripts have seed sequences in 3’UTRs for conserved miR families that can potentially down-regulate the proteins. The seed sequence for the miR-29 family is shown. **B.** 293T cells were co-transfected with a *ETO* 3’UTR-luciferase reporter construct and NS, miR-15 or miR-29 mimics. Cells were harvested 48 hours post-transfection and the luciferase activity was measured. Bar graphs represent two independent experiments, and show that miR-29 targets the ETO 3’UTR as indicated by a significant decrease in luciferase activity (*p* > 0.01) **C.** SKNO-1 cells expressing the indicated miRs were harvested for western blot analysis 5 days post infection. Protein levels for ETO were determined by antibodies from two independent sources (see Materials and Methods for details). Levels of CDK6, a known target of miR-29a, were also examined. Tubulin was used as a loading control. Western blot represents results from two biological replicates.

Since the AML1-ETO 3’UTR has a seed sequence for members of the miR-29 family (Figure 2A), we experimentally tested whether miR-29b-1 targets AML1-ETO. A luciferase reporter carrying the AML1-ETO 3’UTR was co-transfected in 293T cells with a non-silencing miR (NS), miR-15, which also has seed sequence in AML1-ETO 3’UTR, or miR-29. Cells were harvested 48 hours post transfection and the luciferase activity was determined. As shown in Figure 2B, miR-29, but not the NS control or miR-15, significantly reduced luciferase activity (*p* < 0.01), indicating that miR-29 specifically targets the AML1-ETO 3’UTR. We investigated whether miR-29 also targets and down-regulated endogenous AML1-ETO protein. Lentiviruses containing miR-29b-1 or miR-15 (another miR that can potentially target AML1-ETO, and is downregulated in AML patients) were generated and used to infect patient-derived SKNO-1 cells that also carry the t(8;21). A non-silencing control miR (NS) was included as a negative control. We routinely and reproducibly observe more than 80% infection efficiency of SKNO-1 cells (Supplementary Figure 2). Total RNA and protein were collected from infected cells 5 days post infection. Quantitative PCR showed little to no effect on the AML1-ETO transcript in three independent experiments (data not shown)[36]. Importantly, miR-29b-1, but not the NS control or miR-15, specifically down-regulated AML1-ETO protein levels (Figure 2C). Levels of CDK6, a verified target of miR-29 in mantle cell lymphoma [31], remained unchanged, indicating cell type specific downregulation of targets by miR-29 family members. Together, these findings identify the *miR-29b-1* locus as a direct transcriptional target of AML1-ETO and implicate miR-29b-1 in regulation of the leukemic phenotype by downregulating the AML1-ETO protein.

### MicroRNA-29b-1 decreases cell growth and induces apoptosis

**Figure 3:**
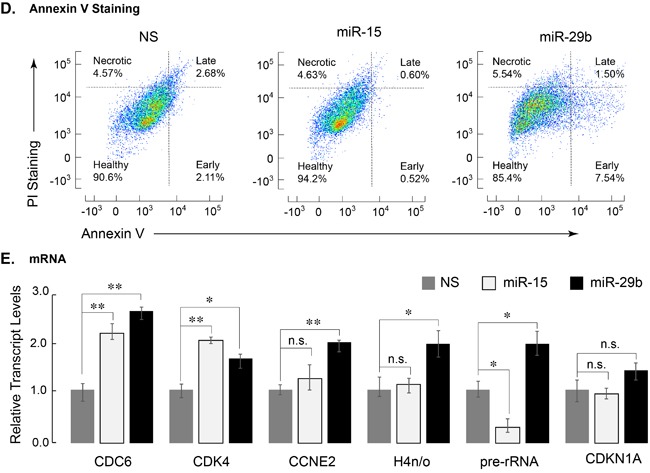
Leukemia cells expressing miR-29b-1 exhibit decreased cell growth/metabolic health, increased apoptosis, and modified expression of cell cycle related genes **A.** SKNO-1 cells expressing indicated miRs were analyzed for NADPH activity. Two independent biological replicates show that miR-29b-1 (black bar) adversely affects metabolic health of SKNO-1 cells when compared to NS control (gray bar) or miR-15 (white bar) (*n* = 4; *p* > 0.01). **B.** Equal number of SKNO-1 cells (2.5 × 10^5^), infected with NS (gray triangle), miR-15 (open square), and miR-29b (closed circle) were plated in triplicate wells in 12-well plates, and counted for live cells each day for 5 days using the trypan blue dye. Line graphs represent two independent experiments. **C.** SKNO-1 cells infected with NS (gray), miR-15 (blue), and miR-29b (red) were stained with propidium iodide and analyzed for cell cycle profile. Arrow indicates accumulation of sub-G1 population in miR-29b-infected cells. **D.** Infected SKNO-1 cells were examined for apoptosis by AnnexinV-Cy5 staining. Cells were counterstained with propidium iodide (PI). Scatter plots of analyzed data are shown and percent of cells in each of the four quadrants indicating healthy, early apoptotic, late apoptotic and necrotic cells is shown. **E.** Expression of cell cycle related genes was determined by qPCR of total cellular RNA from SKNO-1 cells infected with NS (gray bars), miR-15 (white bars) and miR-29b (black bars). Bar graphs represent data from three independent experiments; expression of each gene is normalized to that of *HPRT*. Error bars indicate standard deviation (*n* = 6), and the asterisks show significance *i.e*., * = *p* < 0.05, ** = *p* < 0.01, n.s. = not significant.

It has been shown that shRNA-mediated downregulation of AML1-ETO leads to simultaneous activation of proliferation and pro-apoptotic pathways [10], [37]. We hypothesized that miR-29b-1 down-regulation of AML1-ETO may lead to similar effects on cell proliferation and survival. SKNO-1 cells were infected with NS or miR-29 carrying lentiviruses. Because miR-15 did not decrease luciferase activity in 3’UTR-luciferase assay (Figure 2B) or affect AML1-ETO protein levels (Figure 2C), it was included as an additional control for specificity. Infected cells were harvested 5-days post-infection and were subjected to a variety of assays that are designed to determine cell growth and proliferation, as well as cell cycle parameters and cell survival. Initially, we assessed cell proliferation using the Cell Counting Kit-8, a colorimetric assay to determine cell viability/health by quantifying NADPH activity. As shown in Figure 3A, the CCK-8 assay revealed a significant decrease (*p* < 0.01) in the percentage of healthy cells expressing miR-29b-1, *i.e*., only ~70% of the cells were metabolically healthy. We verified these findings by examining the cell growth post-infection. Equal number of infected SKNO-1 cells (2.5 × 10^5^) were plated in triplicates in 12-well plates, and live cells were counted using trypan blue dye every day for 5 days. Consistent with reduced metabolic health, SKNO-1 cells expressing miR-29b-1 showed reduced cell growth compared to NS or miR-15-infected cells (Figure 3B). We also find that miR-29b-1 expression decreases BrdU incorporation in SKNO-1 cells, indicating an effect on the S-phase and/or DNA repair (Supplementary Figure 3).

We examined whether miR-29b-1 expression had any effect on the cell cycle of leukemia cells. Cell cycle profiles, as determined by propidium iodide staining and FACS, were comparable among the NS, miR-15 and miR-29-expressing cells, although cells expressing miR-29b-1 showed a considerable sub-G1 population, indicating cell death (Figure 3C). We experimentally determined whether miR-29b-1 is activating pro-apoptotic pathways in leukemia cells. Infected cells, stained with Annexin V, a cell surface antigen indicative of apoptosis [38], were analyzed by FACS. In comparison with cells expressing NS or miR-15, miR-29b-1 expressing cells showed a significantly higher percentage at various stages of apoptosis (14.6% in miR-29b-1 vs. 9.4% in NS and 5.8% in miR-15) (Figure 3D). Collectively, these observations show that miR-29b-1 induces apoptotic pathway in these AML cells.

We complemented these observations by evaluating the expression of genes associated with cell cycle regulation. Transcript levels of several genes that included histone and cyclin-dependent kinase genes were determined by qPCR. The S-phase related genes CDC6, CDK4, Cyclin E2, and histone H4n/o showed significant upregulation in cells expressing miR-29b-1. Interestingly, expression of the growth related rRNA genes that are upregulated by AML1-ETO [39] was also increased significantly in cells infected with miR-29b-1 (Figure 3E). Together, these data indicate that expression of miR-29b-1 increases cytotoxicity and apoptosis, decreases BrdU incorporation and upregulates S-phase-related genes, at least in part through down-regulation of AML1-ETO.

### MicroRNA-29b-1 impacts on MPO activity and colony forming capability and partially reverses the AML1-ETO-mediated transcriptional program

**Figure 4:**
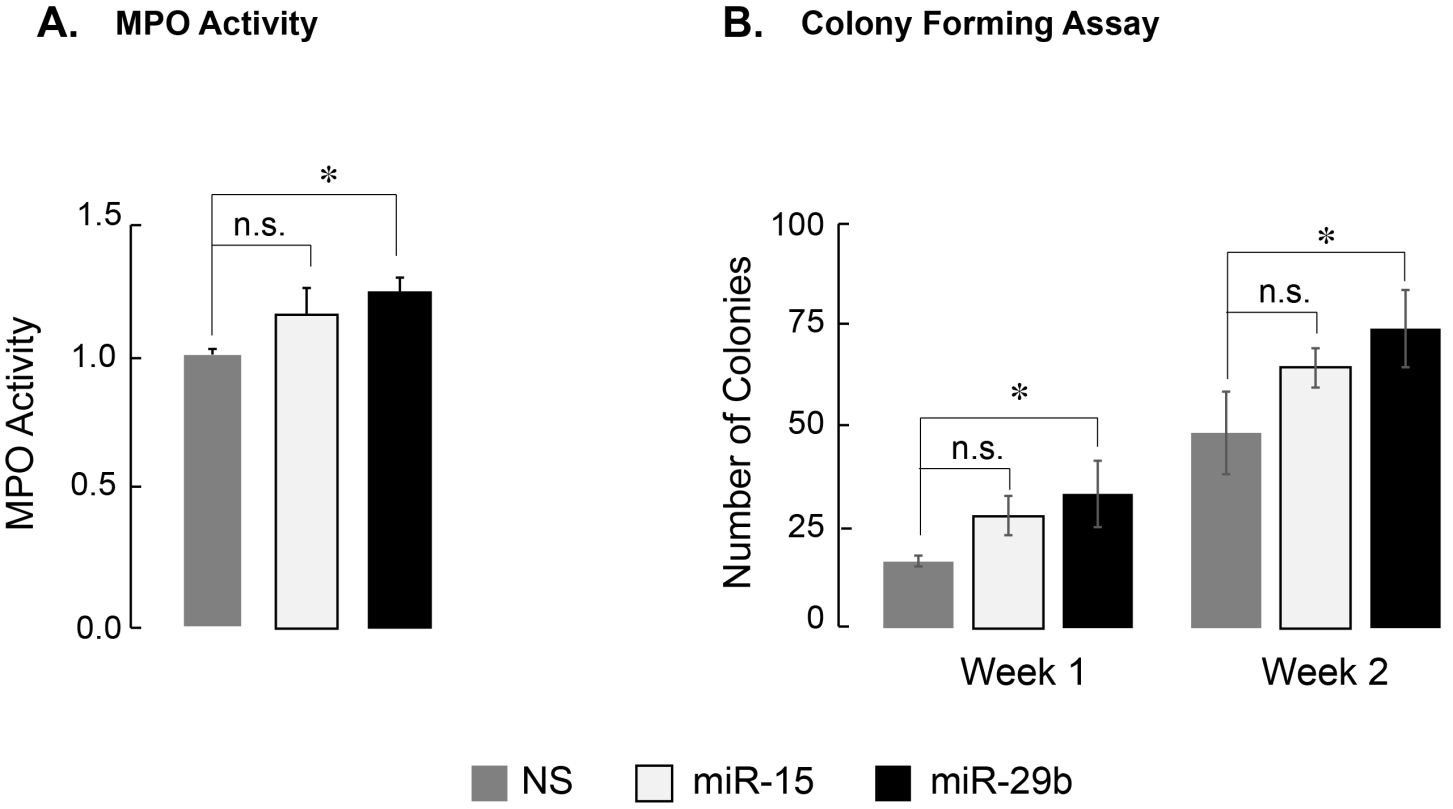
Expression of miR-29b-1 partially relieves differentiation block in leukemia cells **A.** Activity of myeloperoxidase, a marker for promyelocytic differentiation, in SKNO-1 cells expressing indicated miRs is shown. miR-29b-1 causes a modest, but significant increase in MPO activity (denoted by * (*p* < 0.05); n.s. = not significant). Bar graphs are an average of two independent experiments. **B.** Quantification of SKNO-1 colonies in methocult soft medium. Each week, colonies were counted from duplicate plates for each condition, and are shown here as an average of the two counts from two independent experiments. Asterisk (*) denotes *p* < 0.05; n.s. = not significant.

AML1-ETO modifies the RUNX1-dependent transcriptional program that is essential for myeloid cell maturation, resulting in a differentiation block [2]. We examined whether miR-29b-1, which can downregulate AML1-ETO protein, impacts the differentiation block. We experimentally determined two key parameters of myeloid cell differentiation: activity of myeloperoxidase (MPO), which is indicative of early myeloblast differentiation [40], and colony forming capability of cells in methylcellulose, a determinant of differentiation and proliferative potential. SKNO-1 cells, infected with NS, miR-15 or miR-29b-1, were analyzed for MPO activity 3-days post-infection (Figure 4A). Cells infected with miR-29b-1 showed a modest, but significant (*p* < 0.05), increase in MPO activity when compared to those infected with NS; miR-15 also caused a slight increase in MPO activity, but it was not significant. In parallel, colony forming unit (CFU) assay showed that the number of colonies continued to increase for both miR-15 and miR-29b-1 infected cells during Week 1 and Week 2 after plating infected cells in methylcellulose supplemented with cytokines and growth factors (Figure 4B and Supplementary Figure 4). No change in size or shape of the colonies was observed. Collectively, these findings indicate that miR-29b-1 modestly impacts AML1-ETO-induced leukemic properties.

**Figure 5:**
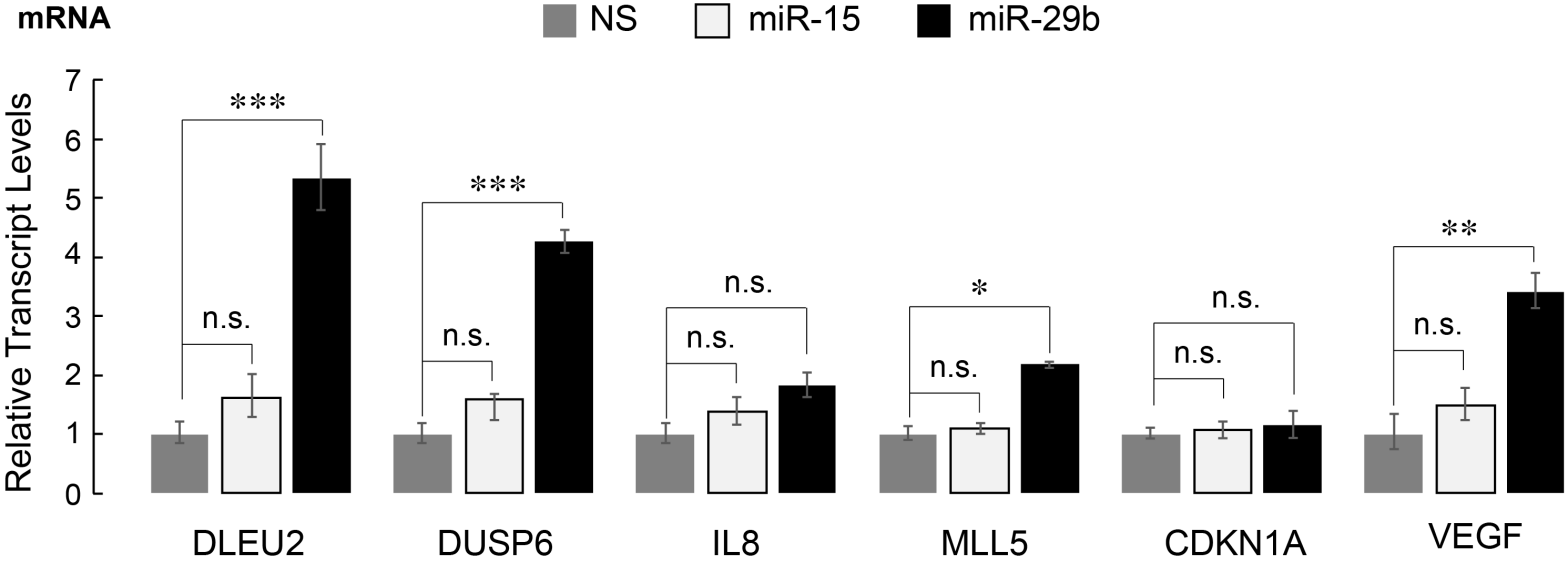
AML1-ETO target genes in leukemia cells are de-regulated upon introduction of miR-29b-1 SKNO-1 cells infected with the indicated miRs were analyzed for expression of several genes that are direct targets of AML1-ETO and contribute to leukemic phenotype [35]. Bar graphs show the average of three independent experiments with each gene normalized to *HPRT*. Error bars indicate standard deviation (*n* = 6); * = *p* < 0.05, ** = *p* < 0.01, *** = *p* < 0.001, n.s. = not significant.

We have previously identified a gene signature that is responsive to AML1-ETO in leukemia cells, exhibits co-occupancy by AML1-ETO and the NCoR co-repressor and is indicative of the leukemic properties associated with AML1-ETO [35]. We hypothesized that miR-29b-1 impacts the leukemia phenotype by modifying the expression of AML1-ETO responsive genes. Expression of a subset of genes that were most responsive to AML1-ETO was assessed by qPCR. Of the genes tested, *DUSP6*, *DLEU2*, *MLL5*, and *VEGF*, all down-regulated by AML1-ETO [41], were significantly upregulated in miR-29b-1-infected cells (Figure 5). In contrast, other known targets of AML1-ETO that include *CDKN1A* and *IL8* [10] were not affected (Figure 5). Together, these findings show that miR-29b-1 contributes to AML1-ETO-driven transcriptional regulation of genes that contribute to leukemic phenotype.

## DISCUSSION

The AML1-ETO oncogenic protein drives the leukemogenic phenotype by disrupting subnuclear localization, protein-protein interactions and the transcriptional program of the key hematopoietic transcription factor RUNX1 [10]. Furthermore, a distinct miR signature is associated with leukemia patients with the 8;21 chromosomal rearrangement [10], [21], [42]. How miRs contribute to AML1-ETO-mediated leukemogenesis remains unresolved. In this study, we identified miR-29b-1 as a regulator of the AML1-ETO protein, and demonstrated that miR-29b-1 expression in t(8;21)-carrying leukemic cell lines partially rescues the leukemic phenotype.

Members of the conserved miR-29 family are directly implicated in hematological malignancies, including AML [30]. For example, miR-29a expression in immature mouse hematopoietic stem cells results in the development of a myeloproliferative disorder. Similarly, over-expression of miR-29b-1 in leukemia cells modifies the expression of genes involved in apoptosis, cell cycle and proliferation pathways. Several targets for miR-29 family members have been validated and include Myc, ABL1, Cyclin D2 and AKT2 [31], [33], [43]. These reports are consistent with our findings that miR-29b-1 targeting of AML1-ETO results in decreased BrdU incorporation and increased apoptosis, as well as changes in expression of genes associated with cell growth (e.g., rRNA genes) and cell cycle progression (e.g., CDC6). Because many of these targets regulate major cellular processes (e.g., Myc), it is likely that several compensatory mechanisms are invoked by miR-29 family members, resulting in modest and partial effects on cell growth, proliferation and differentiation. Importantly, we demonstrate that oncogenic AML1-ETO is a direct and novel target of miR-29b-1, providing a mechanistic link between AML1-ETO-mediated leukemogenic properties and tumor suppressor activities of miR-29b-1. AML1-ETO downregulates C/EBPα, a key hematopoietic transcription factor, which has been shown to upregulate miR-29b-1 locus [29]. Our findings that AML1-ETO also occupies the *miR-29a/b-1* locus and transcriptionally regulates its expression suggest that AML1-ETO regulates miR-29a/b-1 locus both directly and indirectly (via regulation of C/EBPα), indicating that a regulatory circuit between AML1-ETO and miR-29b-1 is operative in leukemia cells to drive tumor properties.

**Figure 6:**
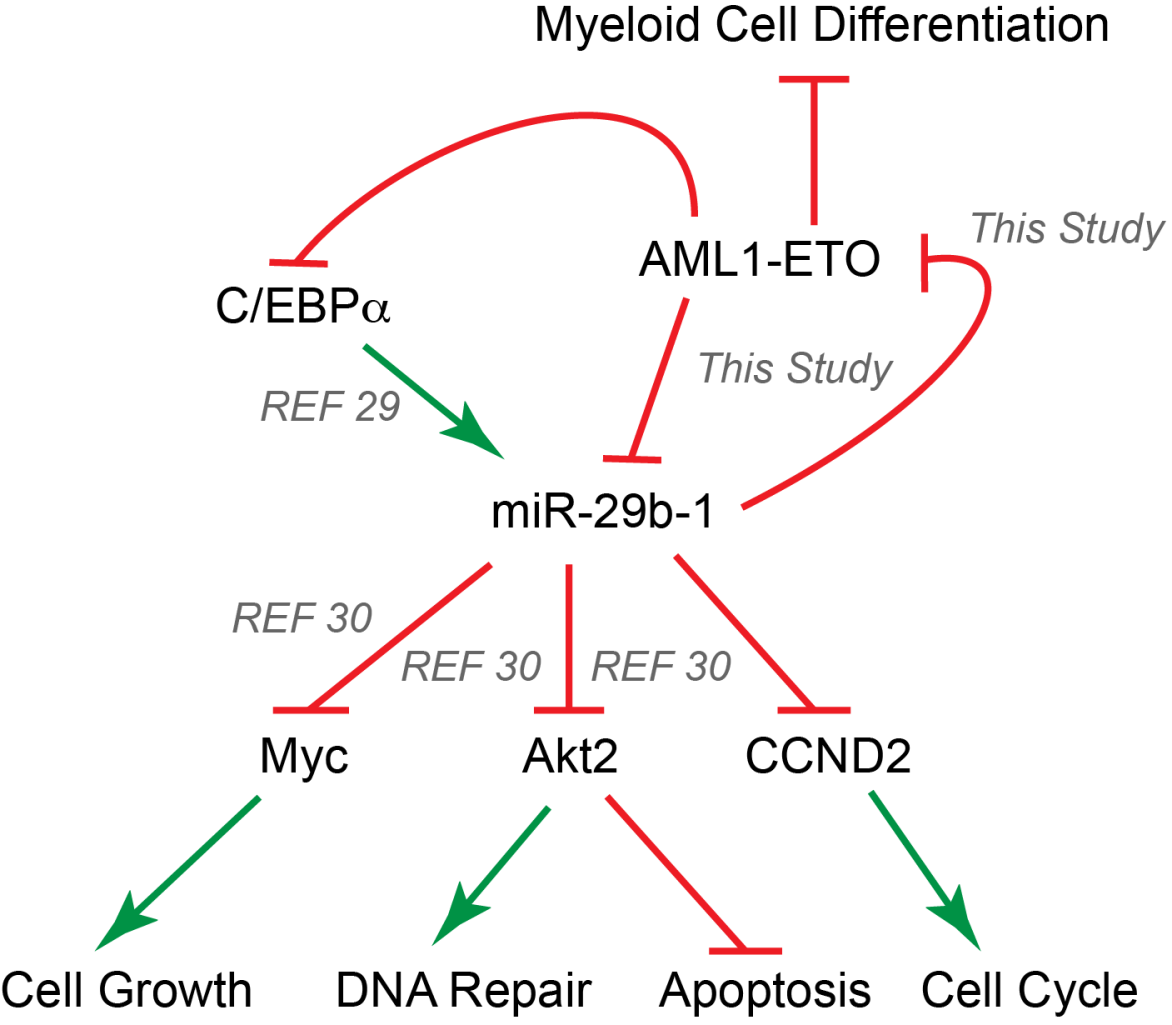
AML1-ETO and miR-29b-1 form a regulatory circuit that modulates leukemic phenotype Our results establish a regulatory circuit between the AML1-ETO oncogene and the miR-29b-1 tumor suppressor; AML1-ETO transcriptionally regulates miR-29b-1, and miR-29b-1 translationally inhibits AML1-ETO. MicroRNA-29b-1 has been implicated in hematological malignancies and is a direct transcriptional target of the hematopoietic C/EBPα transcription factor [29]. Our results suggest that AML1-ETO regulates the expression of miR-29b-1 directly by binding to the promoter regulatory region of the *miR-29a/b-1* locus, as well as indirectly through down-regulation of C/EBPα, which activates the miR-29a/b-1 locus [29]. It has been shown that miR-29b-1 directly inhibits the expression of Myc, a key regulator of cell cycle and cell growth, Akt2, an upstream sensor in the DNA repair/damage pathways and a regulator of cell apoptosis, and Cyclin D2 (CCND2), which mediates cell cycle control [30]. The current study provides evidence of a complex interplay between AML1-ETO and miR-29b-1 that leads to partial apoptosis, decreased BrdU incorporation, and release from AML1-ETO-mediated myeloid differentiation block. Our study and others together implicate miR-29b-1 as a key modulator of leukemic phenotype in acute myeloid leukemia. Green arrows indicate an activating event, while red symbols show an inhibitory event.

Several studies examining a role for the miR-29 family in hematopoietic and leukemia cells suggest non-redundant roles for the family members [25], [30], [43]. MicroRNA-29a promotes hematopoietic stem cell renewal [26], while miR-29b-1 is associated with apoptosis [33]. In this study, we have found a complex phenotype resulting from miR-29b-1 expression in leukemia cells with the 8;21 translocation. Our results shown that miR-29b-1 affects metabolic health and cell growth properties of leukemia cells, induces apoptosis, and modestly increases myeloid cell differentiation and the colony forming capabilities of leukemia cells. Furthermore, miR-29b-1 expression relieves suppression of several AML1-ETO target genes that are directly linked with the leukemic phenotype. These results indicate that miR-29 family members exhibit selectivity which may be cell and/or leukemia type specific. Collectively, we identify a regulatory circuit involving AML1-ETO and miR-29b-1 in leukemia cells. Expression of miR-29b-1, partially reverses the AML1-ETO-dependent leukemia phenotype, thus providing a component of AML1-ETO-mediated control that leads to the onset and progression of disease.

## MATERIALS AND METHODS

### Cell lines and reagents

Kasumi-1 and SKNO-1 cell lines were purchased from the American Type Culture Collection (ATCC, Manassas, VA) and Leibniz Institute DSMZ-German Collection of Microorganisms and Cell Cultures (Braunschweig, Germany), respectively. Cell were maintained in RPMI-1640 media supplemented with 20% FBS. SKNO-1 cells were also supplemented with 10ng/ml recombinant GM-CSF. Following antibodies were used in this study: RUNX1 (Cat. No. 4334, Cell Signaling Technology, Inc., Danvers, MA), ETO (Cat. No. 4498S, Cell Signaling Technology, Inc.), RUNX1T1/ETO (Cat. No. ab124269; Abcam, Cambridge, MA), CDK6 Clone DCS83 (Cat. No. 3136S; Cell Signaling Technology, Inc.), Tubulin Clone DM1A (Cat. No. T9026; Sigma-Aldrich, St. Louis, MO), and normal (Cat. No. sc-2027 for anti-rabbit; Cat. No. sc-2025 for anti-mouse) and HRP-conjugated IgG (Cat. No. sc-2004 for anti-rabbit; Cat. No. sc-2005 for anti-mouse) antibodies (Santa Cruz Biotechnology, Dallas, TX).

### Lentiviral expression, production and infection

Sequences for miR-15 or miR-29b-1 (or scrambled sequence as non-specific (NS) control) were cloned using BLOCK-iT™ Lentiviral Pol II miR RNAi Expression System with EmGFP (Cat. No. K4938-00) from Thermo Fishcer Scientific (Waltham, MA). Lentiviral plasmids carrying non-silencing short-hairpin (sh) RNA or shRNA targeting AML1-ETO (Cat. No. RHS4531-EG862) were purchased from GE Healthcare Dharmacon, Inc (Lafayette, CO). To generate viral particles, 293-FT cell line (Cat. No. R70007; Thermo Fishcer Scientific) was transfected with lentiviral plasmids and envelope-encoding pMD2.G and the packaging vector pspAX2 that encodes for HIV *gag*, *pol*, *rev*, and *tat* genes all from a single packaging vector using Lipofectamine reagent. Medium containing viral particles was collected 48 hours after transfection and viral particles were concentrated using Lenti-X Concentrator (Cat. No. PT4421-2; Clontech, Mountain View, CA) according to manufacturer's protocol. Viral particles were stored at -80°C until use. Five million actively proliferating SKNO-1 cells were infected with an empirically determined volume(s) of viral preparations in the presence of 8mg polybrene per ml of medium and cells were monitored for GFP expression to determine infection efficiency. Infected cells were harvested at indicated times post-infection for various assays reported in this study.

### Chromatin immunoprecipitation and western blot analysis

After 3-5 days of infection, SKNO-1 cells were used for chromatin immunoprecipitation using RUNX1 and ETO antibodies or normal IgG, or subjected to western blot analysis as described [21], [35]. ChIP-seq results were analyzed using established bioinromatics pipeline in our group, and have been published elsewhere [44].

### Quantitative polymerase chain reaction

RNA was isolated as described for microarray analysis, and cDNA was synthesized with random hexamer primers using SuperScript III First Strand Synthesis System (Cat. No. 18080-051; Thermo Fischer Scientific). qRT-PCR was performed using SYBR Green PCR Master Mix (Cat. No. 1725122; Bio-Rad, Hercules, CA); samples were normalized to *HPRT1* and fold change was determined using the ΔΔCt method.

### Cell cycle and proliferation assays

Metabolic health of infected cells was determined using the Cell Counting Kit-8 (Cat. No. CK04-01; Dojindo Molecular Technologies, Inc. Rockville, MD) according to manufacturer's protocol.

### BrdU staining and immunofluorescence

BrdU incorporation assay was performed using BrdU Incorporation kit from Roche (Cat# 11296 736 001) according to manufacturer's protocol. Images were captured with a Ziess Axioplan microscope and processed using the associated ZEN software.

### Annexin V staining and fluorescence-activated cell sorting

Infected cells were subjected to Annexin V staining using AnnexinV-Cy5 apoptosis detection kit from Abcam (Cat. No. ab14150) according to manufacturer's protocol. Cells were counterstained with propidium iodide and analyzed for apoptosis by fluorescence activated cell sorting (FACS) using a BD LSRII analyzer and Flowjo software.

### Myeloperoxidase (MPO) activity assay

Activity of the MPO enzyme was determined using Myeloperoxidase Activity Colorimetric Assay Kit (Cat. No. ab105136) from Abcam (Cambridge, MA) according to manufacturer's protocol.

### Colony forming assay

Colony forming assays were performed using MethoCult™ H4034 Optimum (Cat. No. 04044) from Stem Cell Technologies (Cambridge, MA) according to manufacturer's protocol. Briefly, 5000 cells from each condition were carefully resuspended in MethoCult™ H4034 Optimum and plated in duplicates in 35mm cell culture plates. Colonies were counted each week for two weeks, and are presented as average of two independent experiments (*n* = 4).

## SUPPLEMENTARY MATERIALS FIGURES AND TABLES


