# Supplementary material for: An AML1-ETO/miR-29b-1 regulatory circuit modulates phenotypic properties of acute myeloid leukemia cells
Source: Oncotarget. 2017 May 24;8(25):39994–40005. doi: 10.18632/oncotarget.18127 (PMC5522207; doi:10.18632/oncotarget.18127)
Supplement: Supplementary file 1 [file oncotarget-08-39994-s001.pdf]

An AML1-ETO/miR-29b-1 regulatory circuit modulates phenotypic properties of acute myeloid leukemia cells

Supplementary Material

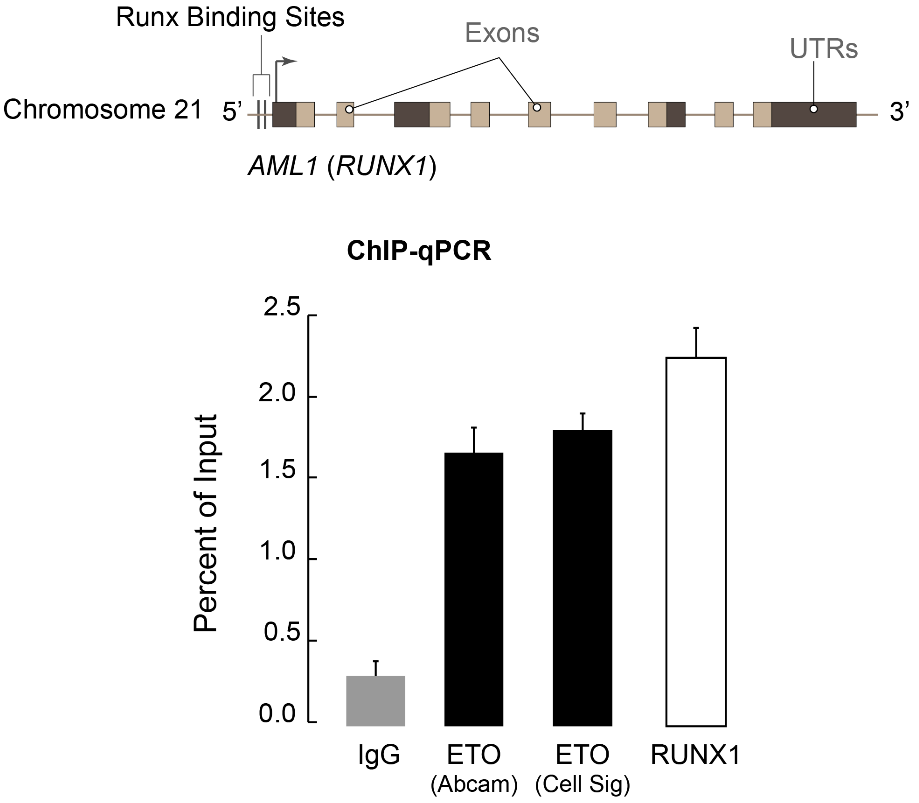

Supplementary Figure 1: Schematic of the *RUNX1* gene, located on human chromosome 21, showing RUNX binding sites, untranslated regions (UTRs) and exons. *RUNX1* gene, which is transcriptionally auto-regulated by RUNX1 and is occupied by both the wild type RUNX1 and the leukemic AML1-ETO protein, was used as a positive control for validation of occupancy of the miR-29b-1 locus by AML1-ETO and RUNX1 (see Figure 2) using chromatin immunoprecipitation-qPCR. Chromatin, immunoprecipitated from SKNO-1 cells using the indicated antibodies, was subjected to qPCR using specific primers surrounding the RUNX binding sites in the P1 promoter of the *RUNX1* gene. As expected, both proteins occupy the RUNX1 P1 promoter (compare the grey bar for the IgG control with black bars indicating ChIP using two different antibodies against the ETO moiety, and white bar indicating ChIP using a RUNX1 antibody).

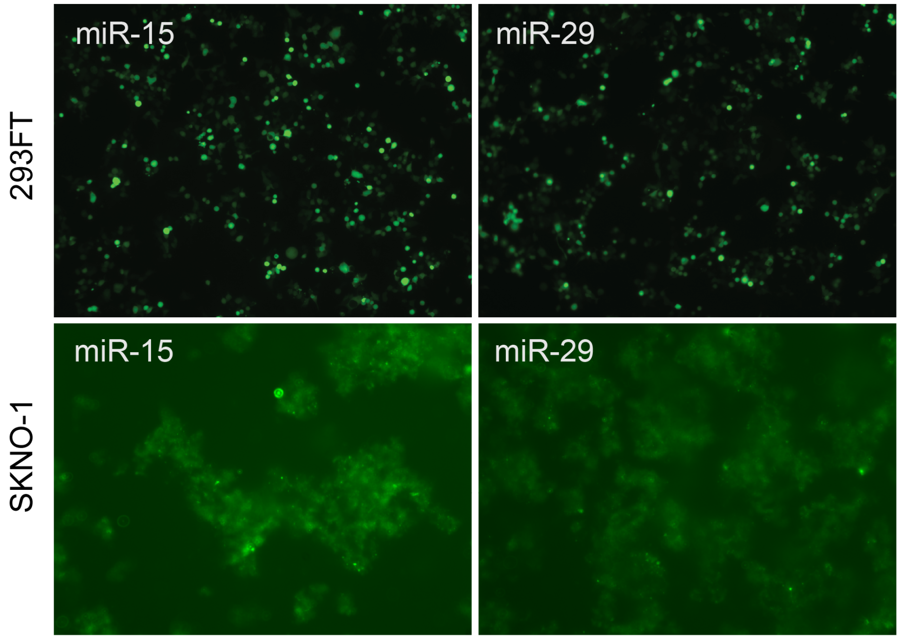

Supplementary Figure 2: Lentiviral production in 293FT cells (images captured 48 hrs after transfection) and infection of SKNO-1 cells (images captured 72 hrs after infection).

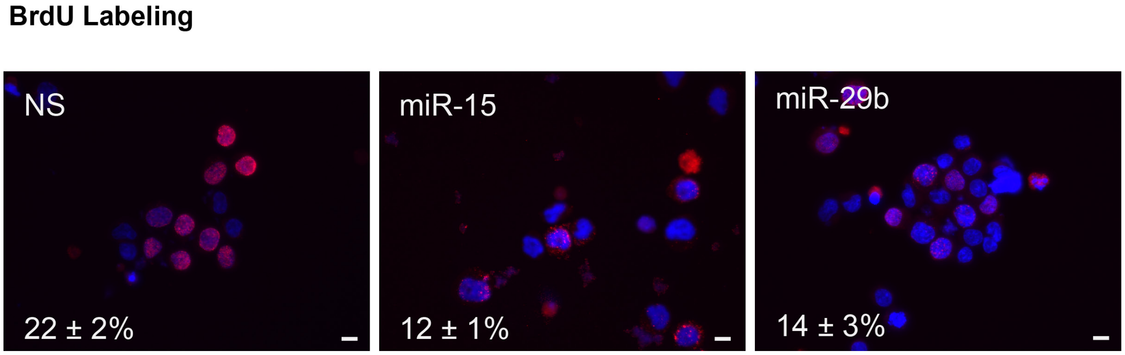

Supplementary Figure 3: Percent of S-phase cells (red) was assessed by BrdU incorporation using immunofluorescence microscopy. Cells were counterstained with DAPI (blue). Two separate coverslips (50 cells each) were scored for BrdU incorporation. Percentage shown represents an average of two independent experiments (total n=4). Scale bar is 10μm. BrdU labelling showed a decreased percentage of positive cells (14±3%) in miR-29b-1 expressing cells when compared to those expressing NS (22±2%); miR-15 also exhibited a decrease in BrdU-labelled cells (12±1%).

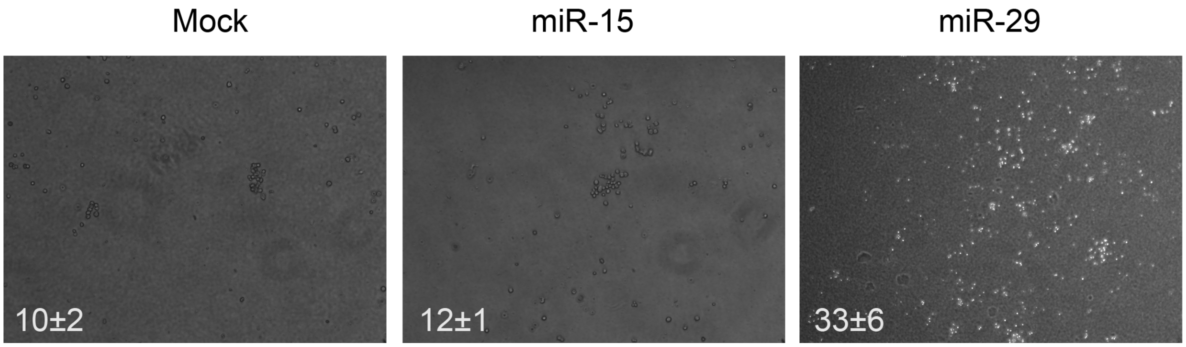

Supplementary Figure 4: Micrographs showing colony forming assay of infected SKNO-1 cells at 14 days of infection.
